# Design of In Vitro Hair Follicles for Different Applications in the Treatment of Alopecia—A Review

**DOI:** 10.3390/biomedicines9040435

**Published:** 2021-04-16

**Authors:** Matej Žnidarič, Žan Michel Žurga, Uroš Maver

**Affiliations:** Faculty of Medicine, Institute of Biomedical Sciences, University of Maribor, SI-2000 Maribor, Slovenia; matej.znidaric97@gmail.com (M.Ž.); zan.zurga@gmail.com (Ž.M.Ž.)

**Keywords:** hair follicle, alopecia, dermal papilla cells, hair transplantation, in vitro hair follicle models

## Abstract

The hair research field has seen great improvement in recent decades, with in vitro hair follicle (HF) models being extensively developed. However, due to the cellular complexity and number of various molecular interactions that must be coordinated, a fully functional in vitro model of HFs remains elusive. The most common bioengineering approach to grow HFs in vitro is to manipulate their features on cellular and molecular levels, with dermal papilla cells being the main focus. In this study, we focus on providing a better understanding of HFs in general and how they behave in vitro. The first part of the review presents skin morphology with an emphasis on HFs and hair loss. The remainder of the paper evaluates cells, materials, and methods of in vitro growth of HFs. Lastly, in vitro models and assays for evaluating the effects of active compounds on alopecia and hair growth are presented, with the final emphasis on applications of in vitro HFs in hair transplantation. Since the growth of in vitro HFs is a complicated procedure, there is still a great number of unanswered questions aimed at understanding the long-term cycling of HFs without losing inductivity. Incorporating other regions of HFs that lead to the successful formation of different hair classes remains a difficult challenge.

## 1. Introduction

Hair is physiologically a part of the integument system, serving as a protective barrier, but it is arguably just as important in its aesthetic function in complementing facial features. Alopecia, even though completely harmless in terms of survival, can have a big impact on the quality of life, particularly on psychological well-being [1]. Nearly 50 million men and 30 million women suffer from hair loss in the US alone. Prevalence values for male pattern hair loss (MPHL) range from 16–96%, depending on whether mild forms of MPHL are included. Interestingly, female pattern hair loss (FPHL) prevalence values are comparable to MPHL [2]. An increasing number of young patients are experiencing signs of alopecia, and only a few effective treatments for alopecia currently exist on the market [2]. Besides pharmacological treatment, surgical hair restoration methods are becoming increasingly popular, with a market value of $8452.5 million in 2018, estimated to rise to $12,119.4 million by 2026 [2]. Over the last few years, the hair restoration industry has been accelerating research towards new trends and innovations. Among these, the follicular unit extraction segment garnered a 34.3% share of the market in 2018 [2]. Currently, the most promising ways to further advance the field of regenerative hair medicine seem to be developing different methods for extracting trichogenic cells to grow small hair-follicle-like structures. These functional hair follicles (HFs), grown in cell cultures, could make the process of hair transplantation easier and prevent the use of animals for hair-related research purposes [2].

The aim of this review is to provide an integrated, synthesized overview of the current knowledge on the design of in vitro HFs for various applications in the treatment of alopecia. Primarily, it focuses on the evaluation of cells, materials, and models for the development of in vitro HFs, emphasizing the evaluation of existing methodological approaches to develop fully integrated applications of in vitro HFs in the field of hair transplantation and pharmacological testing procedures.

## 2. Materials and Methods

A literature review was conducted via the most extensive medical literature databases (Medline, PubMed, ScienceDirect, and Google Scholar) for the timeframe 1995–2021. The search terms used were: “hair loss”, “alopecia”, “in vitro hair follicle”, “dermal papilla cells”, “3D hair follicle culture”, “monolayer hair follicle cultures”, “spheroid hair follicle culture”, “hair follicle induction”, “induction factors hair follicle “, “3D printing hair follicle”, “hair follicle induction”, “dermal papilla expression”, “in vitro hair follicle materials”, “hair transplantation”, “hair reconstructive treatment”. Any relevant review articles, randomized clinical trials, case reports, and case series found were included. In addition to the main research results obtained using the abovementioned databases, references of included resources were further examined and included, if suitable.

## 3. Skin Morphology with Emphasis on the Hair Follicle

**Figure 1:**
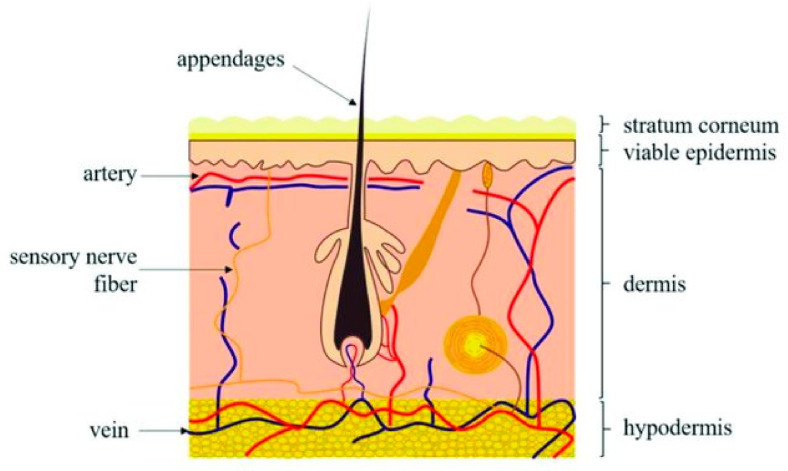
Sketch of human skin with visible three layers of the skin—from the outside to the inside: the stratum corneum (the outmost layer), epidermis, dermis, and hypodermis. Reproduced with permission from [4].

Mammalian skin covers the surface of the body while protecting it from outer environmental influences. As shown in Figure 1, human skin consists of three major layers, the epidermis, dermis, and hypodermis. The epidermis is the first, most superficial layer, composed of the multilayered epithelium, the interfollicular epidermis (IFE), as well as many adnexal structures such as HFs, sebaceous glands, and sweat glands. Depending on location, the distribution of so-called adnexal structures may vary, which is also true for the thickness of the IFE [3].

The hair follicle (HF), often described as a mini-organ, is a complex structure formed with neuroectodermal–mesodermal interaction, which plays an important role in the morphogenesis and growth of HFs [5]. Dermal cells induce hair formation, while epithelial cells respond to the upcoming signal, although the whole process is mutual and complicated [6]. This reciprocal interaction between the epithelium and mesenchyme is controlled by the action of numerous growth stimulators and inhibitors. These lead the development of newly formed HFs through a genetically well-defined procedure involving a series of morphogenic stages, which end with the formation of a fiber-producing mini-organ [7]. Several signaling pathways have been implicated in mesenchyme instruction during embryogenesis and the subsequent dermal papilla (DP) formation. On the other hand, DP-derived instructive signaling is essential to activate epithelial progenitors and initiate HF regeneration and growth. The WNT/β-Catenin pathway is one of the most researched ones due to its role in stem cell differentiation [8,9,10].

Although all HFs vary greatly in shape and size, depending on their location, they are all a constituent of the pilosebaceous unit, along with the sebaceous gland and the arector pilli muscle. The hair follicle can be further subdivided into components of ectodermal origin, consisting of the sebaceous gland and apocrine gland, as well as components originating from the mesodermal mesenchyme of the dermis, which forms the DP, dermal envelope, and cells of the nerve crest, from which the melanocytes are formed [11,12].

**Figure 2:**
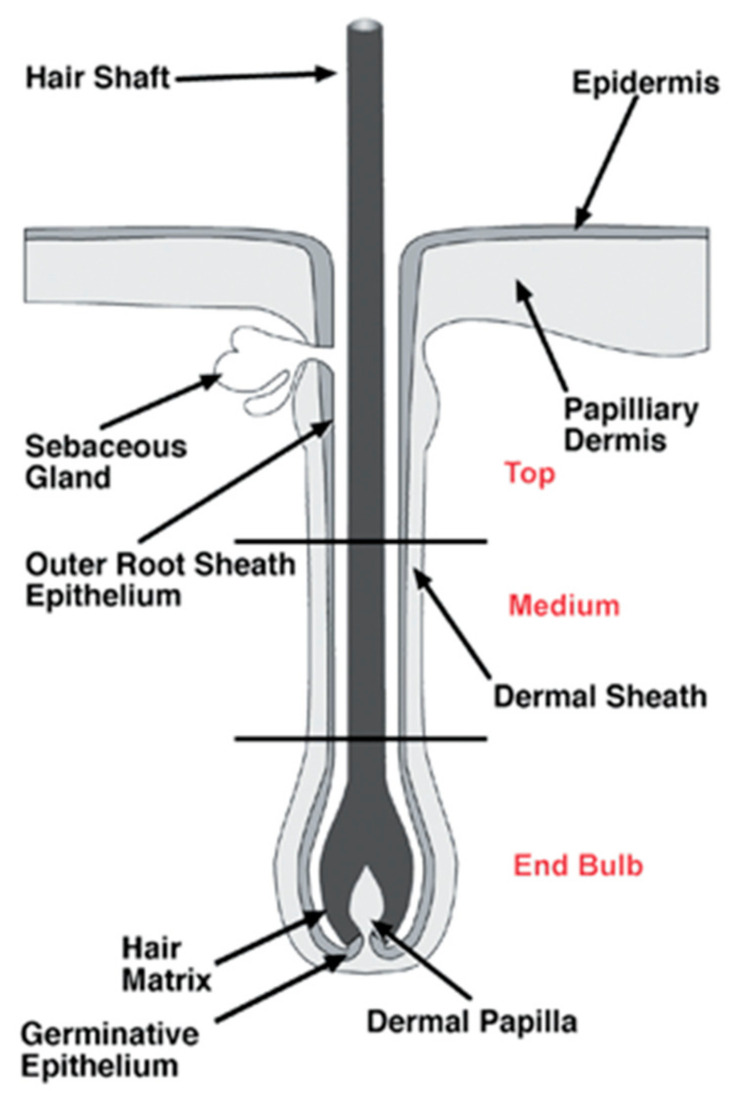
The anatomy of hair follicles (HFs), with the main emphasis on the end bulb and the medium and top sections of the vibrissae, which are separated by black lines. Reproduced with permission from [15].

The structure of HFs, presented in Figure 2, can be divided into three segments: the lower segment (bulge and hair germ (HG)), the middle segment (isthmus), and the upper segment (infundibulum). The HG is located above the DP, a population of mesenchymal cells of the skin in charge of hair growth inductive signals and modulation of HF regeneration, derived from papillary dermal fibroblast lineage [13]. Until recently, it was considered that the bulge was the only reservoir of murine epidermal stem cells, but recent research has shown that other sites of the HF, such as isthmus, infundibulum, and HG, harbor stem cells as well [14].

Upon completion of morphogenesis, the HF perpetually goes through three stages of activity and degeneration, each with its own characteristics [16]. The main factors determining the stage the HF finds itself in are the molecular signals that orchestrate the transition process [7].

### Hair Follicle Cycle

HFs are remarkable for their ability to enter many cycles of regeneration in a lifetime. Still, despite their high regeneration potential, HFs only develop during the process of embryogenesis and, hence, cannot be produced postnatally. This means that any severe injuries or burns affecting the parts containing HFs will cause alopecia [11].

The three stages of the follicular cycle are the anagen phase (marked by regeneration, extremely high proliferation, and protein synthesis activity [17]), the catagen phase (characterized by apoptosis), and the telogen phase (often described as the resting phase). A new hair shaft is formed in each cycle, and the old hair is eventually shed, mostly in an actively regulated process termed exogen [11]. The anagen stage summarizes the start of HF development, when the lower HF is formed with the proliferation of secondary germ cells in the hair bulge [10,18]. The mature anagen HF can be divided into two parts, one that does not cycle visibly, known as the “permanent” upper part, and the lower part, which is continuously remodeled in each hair cycle [18]. Human HFs may remain in anagen for up to 6 years. However, research has shown that patients suffering from alopecia have reduced durations of anagen, decreased HF size, and a higher percentage of HFs in telogen [12,16]. In addition, several months can transpire between hair shedding and regrowth, a lag period that is absent or fleeting in normal individuals [16].

The catagen stage involves the HF’s involution, which is a highly regulated process in which apoptosis of most follicular keratinocytes occurs. Some melanocytes experience the same fate. Follicular melanogenesis also ceases during this stage [19]. Nearing the end of the catagen phase, the DP condenses while moving upward and stops underneath the HF bulge. If the DP fails to reach the bulge during the catagen stage, the follicle stops cycling, and the hair is lost [18]. Current evidence suggests that a few bulge stem cells migrate at the catagen/telogen transition meet the DP, generating the HG [20,21]. Marking the conclusion of the catagen stage is the keratinization of the bottom of the hair fiber, transforming the hair fiber into a club hair, a dead hair possessing a thickened base that anchors it to the HF during telogen [22]. This is the beginning of the telogen stage, during which the HF remains dormant and consists of the bulge and HG. After telogen, the HF is reactivated from the base of the resting structure. The lower hair-producing portion of the HF regenerates, starting the new anagen phase [23].

## 4. Hair Loss

Hair loss (HL), also known as alopecia or baldness, is a common clinical disorder that affects millions of people worldwide and often causes a significant source of patient distress [24]. The Ebers papyrus from 1500 BCE [25] was the first known historical record of alopecia. Since then, numerous studies have been conducted to understand its main causes and pathophysiology better and to find possible treatment methods to stimulate the growth of HFs. The properties of HFs to regenerate and their cyclical growth pattern have become interesting in stem cell biology since HFs have a niche for mature stem cells—hair follicular stem cells (HFSCs). HFSCs are found in the bulge region, which resides in the attachment region of the arrector pili muscles and contains both epithelial and melanocyte stem cells [26].

The diagnosis of HL is accomplished with thorough physical examination and history-taking. Physical examination of patients presenting symptoms of HL should be conducted to evaluate the pattern of HL by examining the scalp and follicular openings with the help of a hand lens or dermatoscopy and, lastly, conducting a hair pull test. Generalized thinning of hair should not be mistaken for HL [27].

**Figure 3:**
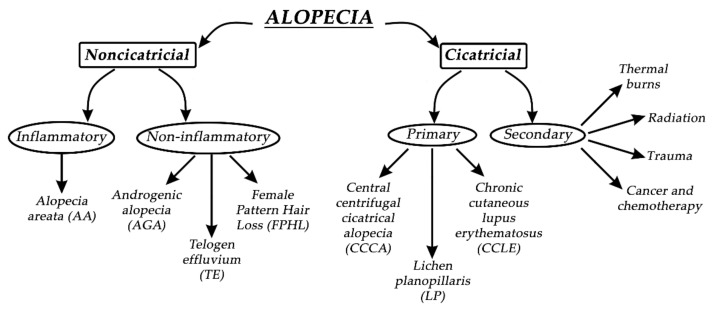
Types of alopecia.

Alopecia, in general, can be divided into two groups—noncicatricial and cicatricial alopecia, as shown in Figure 3. The main difference between noncicatricial and cicatricial alopecia is that cicatricial alopecia is accompanied by scarring, in contrast to noncicatricial alopecia, where scarring is not present [28].

In primary cicatricial alopecia (PCA), HFs are irreversibly destroyed and actively replaced by fibrous tissue [29]. The regeneration of HFs is prevented since the epithelial stem cells are destroyed in the outer root sheath’s bulge at the arrector pili muscle insertion [30]. Secondary cicatricial alopecia (SCA) is mostly caused by cancer and chemotherapy, trauma, radiation, and thermal burns [31]. PCA is further divided into three subtypes: chronic cutaneous lupus erythematosus (CCLE), lichen planopilaris (LP), and central centrifugal cicatricial alopecia (CCCA). Based on The North American Hair Research Society classification, PCA is further divided according to the nature of the predominant inflammatory infiltrate detected in scalp biopsy [32]. Since it is not rare, it represents approximately 7% of patients seen in specialist HL clinics, where one or more patches of permanent alopecia are commonly seen on the scalp. The skin around the active HFs is bald, shiny, and smooth, with closed pores, because of the complete loss of follicular openings [26,32]. The diagnosis of PCA is the most challenging, and the best opportunity to make a definitive diagnosis is to recognize the active inflammation at the site of the bald skin. It is worth mentioning that histological features in the affected sites of bald skin are not specific for PCA, and therefore, clinical examination is more reliable for diagnosis. If alopecia is not diagnosed early, it will result in irreversible HL, with no effective treatment to stimulate the growth of HFs [25]. Therefore, it is crucial to prevent further HL or transplant undamaged donor hair elsewhere on the scalp if the disease is inactive [33].

Noncicatricial alopecia can be divided into inflammatory or noninflammatory types of alopecia. Alopecia areata (AA) is a common psychologically distressing disease, which involves eyebrows, body hair, beard, eyelashes, and scalp; its etiology is closely related to autoimmune conditions such as autoimmune thyroid disease and type 1 diabetes. Due to HL on the scalp region, patients most commonly seek medical help. Patches of lost hair are sometimes light pink; this is caused by inflammation. The pathophysiological origins of AA are closely related to immune response, which specifically targets melanocytic antigens in HFs, and white hairs are commonly spared in patients with grey hair. If the regrowth of hair occurs, it is most commonly white [34]. AA is treated with intralesional corticosteroid injections into the deep dermis of affected regions, but unfortunately, acne and atrophy can result as side effects. Investigational treatments for AA are interleukin-2, interleukin 17, quercetin, JAK inhibitors, and many other active substances, with rather modest effects [35]. Pattern HL is the second most common condition, which independently affects both men and women. By the age of 50, nearly 50% of men develop androgenic alopecia (AGA), which is driven by genetic predisposition and the androgen insensitivity of HFs to androgens (specifically to dihydrotestosterone). Temporal and occipital regions of the scalp are commonly unaffected due to different responses to androgens in different scalp locations. Moreover, hair in AGA does not disappear but becomes short and miniaturized, which visually reflects the appearance of baldness. Female pattern hair loss (FPHL) is a socially distressing cause of HL in the female population, affecting up to 30% of women by the age of 50. It is similar to AGA but most commonly results in hair thinning rather than balding. Hair loss occurs at the crown, with the preservation of the frontal hairline [25,36]. Interestingly, women with FPHL have normal hormone levels. Minoxidil and finasteride are commonly used drugs for treating AGA. Topical minoxidil normalizes HF morphology by increasing the duration of anagen and affecting potassium channels. Finasteride competitively inhibits the enzyme 5α-reductase type II, which blocks the conversion of testosterone to dihydrotestosterone. It is crucial when treating FPHL to evaluate whether androgens are present or not so that the appropriate therapy can be selected [37]. Women are also more affected by telogen effluvium, which is characterized by a diffused hair loss on the scalp. Telogen effluvium (TE) starts with a trigger event 2–4 months before the onset of symptoms, which can be related to an endocrine pathophysiological event, psychological stress, drug-related event, or vitamin deficiency. Acute TE is a self-limited condition, and hair transplantation has no role in the treatment of TE [10]. A recent study has suggested that the stress hormone corticosterone—which is derived from the adrenal gland and is the rodent equivalent of cortisol in humans—regulates hair follicle stem cell (HFSC) quiescence and hair growth in mice. In the absence of systemic corticosterone, HFSCs enter substantially more rounds of the regeneration cycle throughout life. During periods of chronic stress, increased corticosterone levels prolong HFSC quiescence and maintain hair follicles in an extended resting phase [38].

## 5. In Vitro Growth of Hair Follicles

Since the middle of the twentieth century, there has been a strong interest in developing in vitro skin models. From the culturing of 3D human skin biopsies ex vivo to the implementation of 3D printing, there has been a progressive scientific advancement to design skin models in vitro that can mimic human skin morphology and physiology almost entirely [39,40]. Although there have been advancements in bioengineering skin equivalents (SEs), they still do not possess the full complexity of mammalian skin. The complexity of the hypodermis, vasculature, and, especially, hair follicles (HFs) are still not fully understood and, hence, cannot be recapitulated in full detail in the laboratory environment. Current models for HF research are hair follicle organ cultures (HFOCs), monolayer cultures of specific cell types from HFs, 3D coculture systems, human skin transplantation assays, and in vivo reconstitution in immunocompromised mice. Due to legal restrictions in the use of animals in the cosmetic industry (including the 3R principle), there is an increasing need for innovation [39].

Despite all the work done, there is still a need for the development of human HF in vitro cultures to make them a suitable platform for testing various hair-growth-inducing substances and/or transplantation. For such models, the main goal lies in their successful utilization in experiments, aiming to provide previously inaccessible insights into human HF biology and pathology. Furthermore, such models could serve as tools to elucidate our knowledge of signaling within the DP as it relates to induction, maintenance, or even inhibition of hair growth [41].

Comparing 2D cultured dermal papilla cells (DPCs) with intact DPCs and 3D cultures has revealed important differences in morphology, biosynthetic activities, and DPC behavior [42,43]. The problem with monolayer 2D hair follicle models is that they fail to replicate several key features of the HF microenvironment [43]. One of the more crucial drawbacks of 2D cultures is the loss of primary inductive potency of dermal papilla cells (DPCs), which can be seen after a certain period. It predominantly correlates with a loss of the extracellular matrix protein versican and the enzyme alkaline phosphatase at higher passage numbers in the DP [44]. Versican expression in the DP is the highest in the anagen stage, decreased in the catagen stage, and apparently nondetectable in telogen, indicating its importance in the process of maintaining normal HF growth. Patients affected by male pattern baldness were found to have little-to-no versican expression in the DP [45]. It has been proven many times that simple 2D cultures do not show the expression of markers associated with HF inductivity, whereas spheroid-like 3D cultures have the ability to restore the expression of the markers, confirmed by methods such as RT-PCR and immunofluorescence. Miao et al. have examined protein expression in response to DPC cultures on Matrigel-coated plates, representing 3D-like in vitro HF models. Immunoblotting studies showed that adherent DPCs cultured on plastic (2D-style HF model) barely expressed NCAM, versican, and a-SMA proteins, while DP spheroids formed on Matrigel expressed all of them [42,46,47] β-catenin expression was there as well, which is important because of the known fact that the upregulation of canonical WNT–β-catenin signaling is essential for HF growth [48,49,50].

Since 2D models do not offer the characteristics of real hair follicles, most of the studies have put their efforts into developing 3D-like structures that resemble in vivo HFs the most in structure, signaling pathways, and cycling. There are a lot of similarities between the different models, most of them trying to establish and maintain an epidermal–mesenchymal connection to simulate its in vivo counterpart [50]. It seems that one of the simplest and most effective ways to assure this kind of formation is by seeding DPCs into microwells to allow a physiological arrangement of cells in a hair follicle [51]. H.E. Abaci, with his team, used a method of seeding DPCs into microwells, crafted in a way to allow a physiological arrangement of cells in a hair follicle.

**Figure 4:**
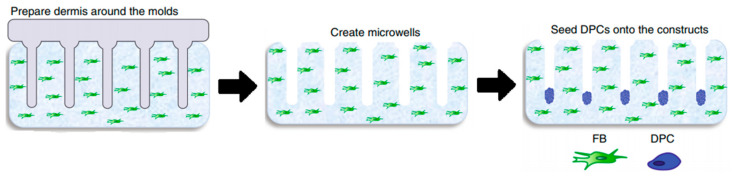
Molds were used to create an array of microwells from the collagen gel containing dermal fibroblasts, in which DPCs were allowed to form spontaneous aggregates. DPCs—dermal papilla cells; FB—fibroblasts. Reproduced with permission from [50].

In order to create microwells of proper dimensions, a mold was crafted with the help of a 3D printing machine. The base of the model in Figure 4, on which the HF-like extensions of the mold are printed, usually consists of one of the molecules building the extracellular matrix (ECM) in vivo, often chosen to be type-1 collagen, either on its own or combined with fibroblasts [50]. However, more appropriate molecules, with fewer limitations, are being researched [50]. Abaci et al. concluded that overexpressing the MR gene *Lef-1* in combination with spontaneous DPC spheroid formation in the HSCs showed a significant increase (from 19% to 70%) in the success rate of ex vivo HF formation compared to empty-vector-transfected DPCs. However, appropriate selection of growth factors, small molecules, and proliferation enhancers needs to be addressed in future studies [48].

The process of forming the perfect HF model that closely resembles the in-vivo-formed HF in all its important characteristics presents a challenge for research teams, presenting them with many problems. Methods using 3D printing technology, particularly 3D printed molds, have shown the most promising results but are still limited by resolution in the spatial control of cells. Adding a stem cell niche, such as the bulge region, is crucial in future research. Using 3D-bioprinters that would operate at single-cell resolution could mean the ability to add stem cells and melanocytes to the HF, allowing in-vitro-formed HFs to be capable of cycling and producing pigment [45].

**Figure 5:**
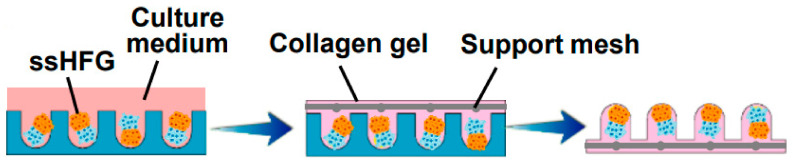
Self-sorted HF germs (ssHFGs) were grown in culture medium and later embedded in collagen gel to enable their transplantation. Reproduced with permission from [48].

Current models also have lots of limitations regarding the materials used, such as collagen-based models, for which the main problem lies in an extensive contraction that may happen after transplantation due to a number of proteinases active in vivo [45]. Another example is Matrigel, the origin of which (it is derived from murine sarcoma cells) causes doubts regarding its adequacy for the development of human-cell-based HF models, despite its reportedly successful use in similar models [51]. The materials mentioned above are two of the most common in vitro models now, but there is an increasing number of ongoing research studies focused on the development of a more favorable model in terms of achieving an optimal environment for human HF growth. One of the newer materials, first used by Gupta et al. in 2018, is cytocompatible silk fibroin protein. Furthermore, a gelatin-conjugated hydrogel has been developed to overcome some of the mentioned drawbacks. The hydrogel was shown to support long-term cell viability in a number of primary and stem cell populations, providing an optimal 3D microenvironment for the immobilization of growth factors, supporting the remodeling of the pericellular matrix and triggering the activation of WNT–β-catenin signaling [44]. Recently a human-derived ECM hydrogel from placenta cells was used to grow HFs in 3D culture, managing to successfully restore the hair-inductive capacity of high-passaged DPCs (shown in Figure 5) [48].

When considering the choice of material used for the base (ECM) of 3D spheroid cultures, it is also important for the surface to be designed in the right way, allowing DPCs to form spheroids. For instance, DPCs cannot form spheroids on the surface of plates pre-coated with a thin layer of Matrigel (2D), but with a thicker coating (3D), the formation of spheroid DPCs is successful. However, it is important to note that some materials do not provide a suitable environment for the formation of spheroids in 3D culture, such as hyaluronan, even though it is a major part of the ECM [13]. Another important feature that should not be disregarded is to assure sufficient oxygen supply, which is critical for proper cell growth, especially when 3D aggregates are prepared within a condensed space with very limited interspaces between them [52].

### 5.1. Cells for the Development of In Vitro HF Models

For purposes of culturing HFs in vitro, researchers have experimented with different cell types that would mimic the actual morphology and physiological environment in human skin tissue. DPCs are a population of mesenchymal cells in the human skin, regulating HF growth and serving as a reservoir of multipotent stem cells [53]. In the 1960s, transplanted DPCs from adult HFs demonstrated a regenerative capacity to induce the growth of new HFs [43]. There is a demand to expand human DPCs in vitro without any impact on their in situ properties. Ohyama et al. were the first to elucidate the molecular structure of noncultured human DPCs. In total, 118 human DP signature genes were identified. Bioinformatics analysis of this DP gene list revealed that WNT, BMP, and FGF signaling pathways were upregulated in intact DPs. The addition of 6-bromoindirubin-39-oxime, recombinant BMP2, and basic FGF to stimulate these respective signaling pathways resulted in the maintained expression of in situ DP signature genes in primarily cultured human DPCs. It was concluded that DPC exposure to the stimulants mentioned above restored DP biomarker expression, which is normally reduced in cultured human DPCs [54]. Havlickova et al. introduced the minimal criteria when designing a human folliculoid in vitro system, supporting the claim that it sufficiently imitates the in vitro situation. One of their claims is that outer root skin keratinocytes (ORSKs) should be physically interacting under 3D conditions and that basement membrane components should be included in the extracellular matrix because the mesenchyme and HF epithelium interact via a basement membrane. Folliculoid 3D systems should be continuously submerged in the culture to mimic the natural environment of human HFs, and the interaction of the HF epithelium and mesenchyme should be supported by fibroblast-contracted collagen type I gel. Furthermore, epithelial HFCs should form concentric cell aggregates to mimic the epithelial tissue compartments in the hair matrix of the HF in vivo. Epithelial HF cells should possess the ability to proliferate HF/ORSK-type keratinization and express a high level of glycogen expression and a low apoptosis level. Minimal proliferation, minimal apoptosis, specific secretory activities, and strong expressions of hepatocyte growth factor (HGF), neural cell adhesion molecule NCAM, and, especially, versican are essential to obtain appropriate interactions between HF mesenchymal cells in such 3D systems [42,45,48,49,50,54,55,56]. Versican is a large chondroitin sulfate proteoglycan molecule, an important initiator of hair regeneration and hair growth maintenance and an inductor of hair morphogenesis. Versican immunoreactivity in DPC is lost when HFs are affected by male pattern baldness [57,58]. When DPCs are removed from the HF microenvironment in human tissue, they lose their ability to induce hair growth. In 2013, DPCs were first successfully grown in three-dimensional papilla spheroids using hanging drop culture systems between separated epidermis and dermis [59]. Since then, further research has been conducted to prove the hypothesis that the transcriptional signature can be partially restored by the growth of DPCs in 3D spheroid cultures [60]. Nonfollicular cell populations have also gained more interest recently due to the scarcity of donor hair. Multipotent dermal progenitors (SKPs) are similar to DPC and have proven clinical relevance for stem cell replacement applications [13]. Unfortunately, their inductive hair potential in the human species remains a mystery [59]. Although human-induced pluripotent stem cells (hiPSCs) also express the ability to generate DPCs and hair follicle stem cells (HFSCs), strong safety standards should be considered [53].

### 5.2. Materials for In Vitro HF Models

Once more, it is worth mentioning that DPCs quickly lose their ability to induce HFs after they are cultured in a culture medium. Furthermore, it is necessary to activate the WNT and BMP signaling pathways, as well as grow DPCs in 3D aggregates and, finally, culture them with keratinocytes to mimic their native in vivo microenvironment [44]. Different 3D models of HFs require scaffolds made mostly from biomaterials. Biomaterials can be implemented as a matrix that supports the encapsulation of dissociated cells or as a supportive scaffold with known rheological properties for designing 3D cell cultures [58]. The organotypic folliculoid 3D system was prepared from collagen I mixed with human dermal fibroblasts (HDFs) and layered, first, with Matrigel and DPCs and, second, with ORSK on the top. When the cell culture was grown in low calcium and serum-free conditions, it represented optimal growth [56]. In another study, DPCs were also cultured in Matrigel medium, which mimics the niche of DPCs [50]. DPCs were cocultured with human extracellular matrix (ECM) proteins such as collagen IV, fibronectin, and laminin [49]. In another study, DPCs and epithelial cells were cultured in collagen–chitosan scaffolds to stimulate epithelial–mesenchymal cellular interactions [61]. New ECM-like compounds are continuously being tested. In a recent study by Tan et al., the authors indicated some interesting outcomes. Using gelatin methacrylate (GelMA)-coated microwells for HF morphogenesis resulted in the production of heterotypic aggregates over prolonged culture duration, without exfoliation of keratinocytes [62]. 3D-printing technology was also implemented, where plastic molds were microfabricated and used to create an array of microwells on a type I collagen gel containing dermal fibroblasts. DPCs were seeded over microgels, which led to spontaneous aggregate formation. With adjustments to the diameter of the microwells, they controlled the size of the DPC aggregates [48]. In 2019, a systematic approach was introduced to produce 3D core-shell heterotypic spheroids, implementing DPCs, keratinocytes, and HDFs into microarray hydrogels fabricated from poly(ethylene glycol) diacrylate (PEGDA) with the application of soft photolithography [45]. Another study concluded that when DPCs and keratinocytes are compartmented in a core-shell structure and both seeded on an ethylene vinyl alcohol (EVAL) surface, they express the ability to grow into multicellular spheroids [63]. Kageyama et al. presented a method to grow epidermal and mouse/human mesenchymal cells in microwells of a custom-designed array plate, fabricated using gas-permeable PDMS. Spontaneous hair follicle germ (HFG) formation in vitro was observed, which presented a suitable method for large-scale production of HFG cells [64]. A more sophisticated HF organoid model was developed by encapsulating the DP spheroid with HF keratinocytes and HF stem cells in a silk–gelatine hydrogel. A hypoxia-induced medium enhanced DP-specific gene expression and cellular proliferation [65].

## 6. Application of In Vitro Hair Follicles

### 6.1. In Vitro Models and Assays for Evaluating Effects of Active Compounds on Alopecia and Hair Growth

**Figure 6:**
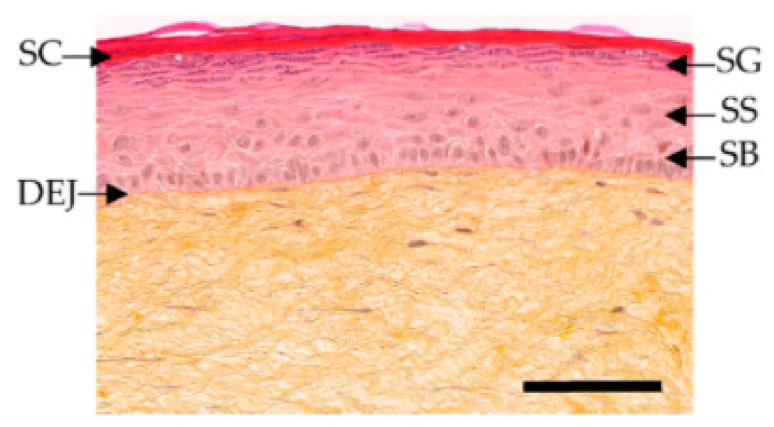
Histological stained images of T-Skin™, with stratum corneum (SC), stratum granulosum (SG), stratum spinosum (SS), stratum basale (SB), and dermoepidermal junction (DEJ). Reproduced with permission from [66].

Since 2004 (the restriction was amended in 2009), animal testing in the European Union has been prohibited for cosmetic ingredients and products. In addition, cosmetic products containing ingredients tested on animals have been forbidden since the year 2009. Since then, many in vitro models to study cosmetic ingredients have become available, serving as valuable and effective tools for testing the absorption and permeability of cosmetic ingredients. For instance, EpiSkin^TM^, developed by L’Oreal, has been validated and recognized as a screening and replacement model. A histological stained image of T-Skin^TM^, with all of its components, is presented in Figure 6.

This model represents the skin’s epidermis layers, mimicking native tissue epidermal layers: stratum basale, stratum spinosum, stratum granulosum, and stratum corneum. It consists of a dermal substrate based on a type I bovine collagen matrix, which represents the dermis. A stratified differentiated epidermis is laid upon it after 13 days in culture. In vitro toxicology tests for dermal corrosivity, skin irritation, and phototoxicity can also be achieved with Epiderm^TM^, SkinEthics^TM^, and epiCS^®^. Possible skin sensitization, also known as contact allergic dermatitis (ACD), can be tested with the direct peptide reactivity assay (DPRA) KeratinoSens^TM^. Systemic toxicity, carcinogenicity, genotoxicity, reproductive toxicity, and endocrine toxicity should also be tested in vitro accordingly [17,41].

Hair-growth-stimulating active compounds have been tested both in vivo and in vitro [67]. In vivo tests provide a better understanding of the pathophysiological processes involved in alopecia. However, due to legal restrictions, amendments to the European Union’s Cosmetics Directive have phased out animal use in testing for any acute toxic effects of beauty products and toiletries [64]. There are different arguments regarding the inappropriateness of animal models, besides the ethical ones. For example, human HF growth phases differ in comparison to animals. Secondly, human HF growth does not occur in a synchronized manner. Therefore, in vitro human HF models to study hair growth are essential to screen for active compounds and test their efficacy for treating alopecia or simply improving the rate of hair growth. On the other hand, there is a scarceness of donated HFs, mainly due to the invasive extraction methods and a limited number of HFs [45].

Since DPCs regulate HF growth and cycling, they are commonly used to study hair growth and regeneration. Because of their short life span in vitro, Kwack et al. developed an immortalized human DPC line, SV40ThTERT-DPC, by introducing the human telomerase reverse transcriptase (*hTERT*) gene into the transformed cell line, simian virus 40 large T DPC (SV40T-DPC) [68]. Following the first study, they cotransfected the SV40T antigen (SV40T-Ag) and hTERT into DPCs from male scalp HFs with AA and established five immortalized DPC lines [69]. Primary and immortalized DPCs are already commercially available. Since HF growth is intertwined with the activity of numerous growth factors (VEGF, IGF, HGF, KGF, TGF-β2) and signaling pathways (ALP, WNT/b catenin pathway, Akt and MAPK, JNK, ERK), the evaluation of DPC proliferation is challenging. A comprehensive review article was published in 2018. Numerous active compounds for HF growth tested on DPCs were evaluated, and their efficacy was later studied in vivo and ex vivo. Active compounds that stimulate DPCs in vitro are minoxidil, numerous herbal extracts, plant actives, natural products, growth factors, cytokines, platelet-rich plasma, placental extract, stem cells, conditioned medium, peptide/proteins, squarticles, and mimetics, among others. Effects of light, electromagnetic fields, and electrical stimulation also induce HF proliferation and stimulate DPCs in vitro. Testosterone and DHT exert inhibitory effects on DPCs. The assessment of the stemness and senescence properties of DPCs has also been taken into consideration [70].

Although DPCs present an established method for assessing hair growth, they do not resemble the whole HF morphology. Existing assays with DPCs should be cocultured with other HF cells, such as keratinocytes and fibroblasts, to understand better the mechanisms of action of selected active compounds, which might possess the ability to induce the growth of HFs. One recent study by Tze Chiun Lim et al. shed some light on whether DPC-only models are sufficient to investigate different drug effects on HFs. While they do not offer the possibility of examining underlying epithelial–mesenchymal interactions, they might have the potential for the screening of molecules that affect hair growth [71].

Gupta et al. tested three-dimensional DP spheroids with and without a silk–gelatin microenvironment and used them as a screening assay by using a standard drug for AA—minoxidil. DP spheroids were able to express DP-specific genes, which resulted in enhanced ECM production compared to monolayer cultures. Furthermore, they established an in vitro 3D organoid model, where they encapsulated DP spheroids in silk–gelatin and combined them with keratinocytes and stem cells. Elevated HF markers, with epithelial–mesenchymal crosstalk, were established, which provides new insights into understanding cell–cell interactions and mechanisms responsible for HF cycling in vivo. Their 3D HF assay can be used to screen and evaluate different molecules that might represent inductive HF growth properties [67].

Human HFs can be switched from anagen to catagen in vitro but modeling the human hair growth cycle in vitro is impossible. Therefore, the best model to study HF cycling is the murine model [46]. The screening models for hair growth assessment are in vivo C57BL/6 and C3H mice models. Ex vivo human and mouse HF cultures are also commonly used to induce the anagen growth phase of HFs and achieve hair shaft elongation [69]. HF research has been conducted, so far, on mice, rats, hamsters, rabbits, and sheep in laboratory conditions [37]. The widely used de novo model for testing hair growth is the silicone chamber assay, where cells are implanted inside grafting chambers onto the backs of nude mice. Epidermal and dermal cells from newborn mice serve as a positive control. Three weeks after grafting, an area of visible hair can be seen at the site of implantation. When using cells from newborn mice to serve as a positive control, an area of visible hair develops in each chamber at about 3 weeks after grafting. Either one or several cell components can then be replaced with candidate cells. Since the silicone chamber assay requires a surgical procedure, with the help of a special apparatus, and since it is labor-intensive, it is not efficient in high throughput screening procedures but rather used in testing candidate molecules and cells. Furthermore, it can be used for evaluating the effects of specific gene products and their hair inductive abilities in vivo [37].

**Figure 7:**
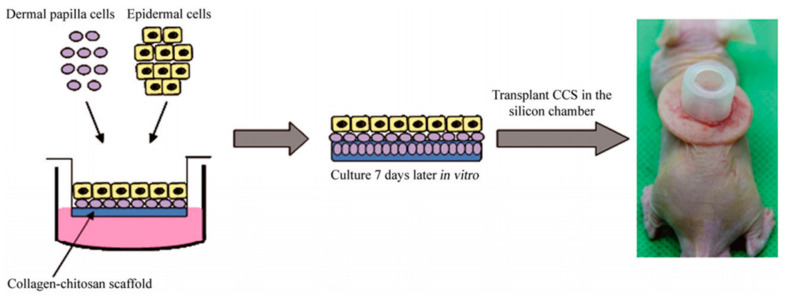
Schematic representation of inoculated DPCs and epidermal cells being transplanted in the silicon chamber on the back of a nude mouse. Reproduced with permission from [56]

Another assay method has been proposed by Zheng et al., where epithelial and mesenchymal cells are injected at certain rations into mice’s hypodermis [37]. K15-positive adult epidermal cells from transgenic mice were isolated, and their ability to generate hair was further evaluated by combining them with neonatal dermal cells in the patch assay. Furthermore, genetic analysis of HF stem cells exposed several known and unknown receptors and signaling pathways that are important for maintaining stem cell phenotypes. Such research could provide potential targets for treating hair loss and other disorders related to hair and skin [37]. Figure 7 shows a similar approach of inoculated DPCs and epidermal cells being transplanted in a silicon chamber on the back of a nude mouse. As with other types of assays, the patch assay also has some limitations, such as difficult visual interpretation of hair growth, which requires a surgical biopsy, and the loss of macro-environment since the orientation of HFs is random [48] The creation of HFs in vitro was recreated in tissue culture, and the proposed method was capable of producing a well-defined hair shaft and revealed similarities in terms of their in vivo counterparts. The assembly of follicular keratinocytes, melanocytes, and fibroblasts was also studied by electron microscopy and molecular analysis. De-novo-created human microfollicles were implemented in existing chip-based human skin equivalents for substance testing. Functional neopapillae or DP condensates needed more than 48 h to form, and after adding keratinocytes and melanocytes, the microorganoids started to self-organize by generating sheath formations, polar segments, and finally, a hair-shaft-like fiber [72].

The ideal model system for a precise understanding of the biochemical function of molecules should, therefore, be able to induce de novo HFs or be able to reconstruct them from their component cells [72]. Further improvements in designing HFs in vitro might establish a gold-standard screening assay and provide an opportunity for creating improved implants for treating wound conditions and transplanting hair in patients with alopecia.

### 6.2. Applications of In Vitro Hair Follicles in Hair Transplantation

Hair loss (HL) in response to various factors and pathological mechanisms such as age, genetics, hormonal imbalances, medical procedures, and trauma is a common disorder affecting both men (about 50%) and women (approximately 25%) worldwide by the age of 50. Consequently, there is a huge demand for effective treatment [50,73]. Currently, the most obvious choices to cure different types of alopecia are pharmacological or surgical treatments. However, they are far from being the ultimate solution since they all lack both efficacy and applicability. Pharmaceutical treatment, being useful only in the early stages of alopecia, brings many negative side effects, especially in the long term, making it anything but ideal. Treatment with minoxidil (Rogaine) can cause scalp irritation and unwanted hair growth on the face and hands. Finasteride (Propecia) can cause diminished sex drive and sexual function and an increased risk of prostate cancer. [74]. On the other hand, surgical treatment has proved itself useful in most cases of hair loss, but in addition to being an invasive method, it has plenty of drawbacks. The two most reliable surgical options are autologous single follicles or follicular unit transplantation. However, the limited number of donor follicles is a big disadvantage. It often occurs that there are not enough available HFs to cover the patient’s alopecic regions, limiting the number of patients suitable for this kind of treatment [74]. Scientists are still trying to develop methods that would be more clinically applicable in terms of invasiveness, cost, technical complexity, and reproducibility and are constantly testing new innovative solutions.

In the case of reconstructive medicine, where larger defects are present together with full-thickness skin loss (not just HF loss by conditions such as androgenic alopecia and alopecia areata), the use of human skin constructs (HSCs), also known as dermal–epidermal composites (DEC), is becoming a new promising approach. They are usually comprised of dermal fibroblasts embedded in a matrix such as collagen, overlaid with keratinocytes [50]. The most prominent problem with HSCs is achieving a full thickness of skin, meaning the presence of the subcutis alongside the formation of complete appendages, also involving mature-shaft-producing hair follicles associated with sebaceous glands [48,59]. An important fact that was discovered is that successful appendage formation is correlated with the donor cells’ age. Wu et al. discovered that HSCs with hairs formed from foreskin epidermal cells (FK Epi), together with fetal dermal (fDer) cells, showed that 15 out of 18 constructs produced hair. On the other hand, HSCs with no hairs formed from adult epidermal cells (Adult Epi) with adult dermal (Adult Der) cells showed that 0 out of 9 constructs produced hair. The results were observed 3 months after implanting HSCs on immunodeficient mice [75]. Through the evolution of 3D bioprinting and robotic harvesting methods, it is expected that we will be able to produce HF-rich HSCs resembling skin, with all its components [59,76,77].

Hair regenerative medicine seems to be the most promising approach for the future. Nevertheless, it is held back by some limitations, particularly problems regarding the development of fully functional transplantable hair follicles of human origin grown in vitro [78,79]. The goal for cell-based regenerative medicine is to be autologous, avoiding tissue immune rejection and, at the same time, restoring epithelial–mesenchymal interactions, imitating the embryonic process of hair follicle neogenesis [48,50,80]. During the anagen phase, the hair follicle is found to be the site of relative immune privilege, expressing a low amount of major histocompatibility (MHC) class Ia antigens and locally producing potent immunosuppressive agents such α-MSH and TGF-β1. The latter could be an indication that the use of allogeneic cells could also be a suitable option for HF regeneration [48]. Recently, it was shown that DPCs are not the only ones able to induce hair follicle formation. Namely, multipotent skin-derived precursors (SKPs) have a similar ability, exhibiting long-term proliferation potential when being cultured in spheroids. Current studies were executed only in mice, but SKPs have been able to induce de novo hair genesis, showing potential for future application of SKPs in hair follicle regeneration and bioengineering. Nevertheless, due to potential epigenetic instability, SKP cells may lose their inductive potential after culture expansion. Ling Guo et al. discovered that the supplementation of trichostatin A (TSA) upon culture expansion of SKPs caused an important increase in their hair induction ability by upregulating the level of histone H3 acetylation in K9 and K14, as well as elevating BMP signaling activity [81]. Additionally, other cellular sources with the ability to differentiate into hair follicles are being tested to determine their applicability to the formation of in vitro hair follicles, appropriate for hair transplants [56].

As of now, there have not been any reported cases of in vitro hair follicle transplantation onto the human scalp, but plenty of studies are researching the integration of human cell hair follicles onto the back or scalp of nude mice. Promising results were noticed in studies testing culture array chips that ensure a dense and spatially arranged manner of hair follicles, replicating the density of HFs found on the human head [82]. Kageyama et al. developed a method for large-scale preparation of HFs. After the formation of HFs, they were poured through mesh reinforced collagen to ensure successful removal from the chip. However, upon transplantation, there was a severe contraction of collagen caused by enzyme activity, resulting in the spaces between the hairs shrinking to a tenth of the original, making the collagen base an insufficient option for in vivo transplantation. Even with its drawbacks, the mentioned method can still be a viable option for producing large amounts of transplantable HFs in the future if the collagen is replaced with more optimal material. The study incorporated a mixture of human and murine cells in the experiment, preventing the proper assessment of this method’s feasibility in human HF transplantations and still leaving some room for further experimentation with human-origin trichogenic cells [83].

When extracting only DPCs and expanding them in culture, the problem is losing their inductive potential and the variable quality of the hair shaft. To preserve their ability of induction, specific factors can be added, such as WNT-CM [50], keratinocyte-conditioned medium [42,49] basic FGF Lef-1 overexpression [46,49], or growing them in 3D cultures [83]. Kageyama et al. discovered that the presence of activated platelet-rich plasma releasate (PRPr) provides greater levels of follicular gene expression while maintaining the spontaneous formation of dumbbell-like HFGs. PRPr, containing various signaling molecules, including growth factors and cytokines, also significantly improved hair regeneration ability upon intracutaneous transplantation, suggesting possible benefits for use in hair regenerative medicine [84].

The perfect transplantation method for DPCs has yet to be developed, despite all the efforts in this field. Aoi N. et al. tested five different transplantation methods on nude mice, using either DPCs or fully differentiated dermal papilla tissue (DPT). One method, particularly the hemivascularized sandwich (HSV) method, where the epidermis with the upper part of the dermis was detached, showed promise. The upper dermal fragment was discarded, and the DPT or DPCs were sandwiched between the remaining parts of the dermis and epidermis, which were sutured at the end. Although the epidermis was fixated, the dislocation of DPCs was present to some degree. This method displayed high hair regeneration efficiency compared to the other four methods studied, mainly due to the direct contact of DPCs with the basal layer and sufficient vascularization of the recipient bed. It further shows the great importance of oxygenation in the induction of hair formation by DPC transplantation [83]. A summary of the most interesting available HF models is shown in Table 1.

## 7. Concluding Remarks and Outlook

In the future, the main goal of regenerative hair medicine will be to develop a method that will focus less on the use of follicular unit transplantation surgery and, instead, take advantage of new technologies. As such, it should focus on designing robust large-scale systems, producing more significant amounts of in vitro hair follicles with all the necessary characteristics. Among these characteristics are long-term cycling without losing inductivity and the incorporation of other regions of HFs (melanocytes to form pigmented hairs), which leads to the successful formation of different hair classes. All the mentioned evidence points towards the necessity to develop and use human-HF-based in vitro models with increased complexity, recapitulating their in vivo counterparts in as much detail as possible.

## Figures and Tables

**Table 1:** Table of established in vitro models for hair follicle research.

Model	Main results	Advantages	Limitations	Reference
Spheroid-like 3D cultures	Expression of β-catenin	The ability to restore expression of the markers confirmed by methods such as RT-PCR and immunofluorescence.	Higher costs	[42,46,47]
Monolayer 2D hair follicle models	Adherent DPCs cultured on plastic (2D-style HF model) barely expressed NCAM, versican, and a-SMA proteins	Ease of use, lower cost, and abundant scientific literature surrounding its use	Loss of primary inductive potency of dermal papilla cell (DPC); do not show expression of markers associated with HF inductivity	[43,44]
Animal models	The best model to study HF cycling	A better understanding of pathophysiological processes involved in alopecia	Ethical limitations, human HF growth phases differ in comparison to animals, and growth does not occur in synchronized manner	[45,46]
DPC in microwells	Overexpressing the MR gene Lef-1 in combination with spontaneous DPC spheroid formation in the HSCs showed a significant increase (from 19% to 70%) in the success rate of ex vivo HF formation compared to empty vector-transfected DPCs.	A physiological arrangement of cells in a hair follicle	Difficulties with appropriate selection of growth factors, small molecules, or proliferation enhancers; limitation by resolution in the spatial control of cells	[50]

## Data Availability

All data related to this study is already included in the manuscript text.

## Abbreviations

The following abbreviations are used in this manuscript:

HF	Hair follicle
MPHL	Male pattern hair loss
FPHL	Female pattern hair loss
IFE	Interfollicular epidermis
DP	Dermal papilla
DPC	Dermal papilla cell
DPT	Dermal papilla tissue
HG	Hair germ
HL	Hair loss
PCA	Primary cicatricial alopecia
SCA	Secondary cicatricial alopecia
CCLE	Chronic cutaneous lupus erythematosus
CCCA	Central centrifugal cicatricial alopecia
AA	Alopecia areata
AGA	Androgenic alopecia
SE	Skin equivalent
HFOC	Hair follicle organ culture
ECM	Extracellular matrix
ssHFG	Self-sorted hair follicle germ
ORSK	Outer root skin keratinocyte
hiPSC	Human-induced pluripotent stem cell
SKP	Skin-derived precursor
HFSC	Hair follicle stem cell
HDF	Human dermal fibroblast
PEGDA	Poly(ethylene glycol) diacrylate
EVAL	Ethylene vinyl alcohol
ACD	Contact allergic dermatitis
DPRA	Direct peptide reactivity assay
hTERT	Human telomerase reverse transcriptase
HSC	Human skin construct
DEC	Dermal–epidermal composites
MHC	Major histocompatibility class
TSA	Trichostatin A
HSV	Hemivascularized sandwich
